# Automatic Data Reduction of Image Sequences Acquired in Object Tracking Mode for Detection and Position Measurement of Faint Orbital Objects

**DOI:** 10.3390/s26051628

**Published:** 2026-03-05

**Authors:** Radu Danescu, Vlad Turcu

**Affiliations:** 1 Computer Science Department, Technical University of Cluj-Napoca, Str. Memorandumului nr. 28, 400114 Cluj-Napoca, Romania; 2 Astronomical Observatory of Cluj-Napoca, Romanian Academy Cluj-Napoca Branch, Str. Ciresilor nr. 19, 400487 Cluj-Napoca, Romania; vladturcu@acad-cj.ro

**Keywords:** space surveillance and tracking (SST), data reduction, image processing, precise object tracking

## Abstract

**Highlights:**

**What are the main findings?**

- A method for complete automatic processing of images acquired in precise object tracking mode, based on lightweight image operations and freely available calibration tools.
- Accurate measurement of the position of faint orbital objects.

**What are the implications of the main findings?**

- Fast reduction in image sequences using low processing power can generate result tdm files at the observer site immediately after the observation is completed.
- Helpful for surveying high altitude or high eccentricity objects, especially on time critical campaigns such as reentry events.

**Abstract:**

Precise object tracking of space objects is an image acquisition method that uses the mount of the telescope to orient the instrument in real time towards the target to be tracked, compensating for the target’s motion. Using this method, the object of interest will appear as a circular or point-like shape in the acquired image, while the background stars will appear as streaks. Using precise object tracking, the light from a faint object accumulates in the same region of the image, increasing the chance of observation, but longer exposures also increase the length of the background star streaks and makes the astrometric calibration difficult. This paper presents a method for the automatic processing of image sequences acquired in precise object tracking mode. Our proposed method includes a filtering mechanism that will ensure local maxima in the center of star streaks in order to allow for a publicly available astrometric calibration software to work even if the stars are not point-like, a weighted stacking mechanism to increase the signal-to-noise ratio for faint targets while excluding the stars, an automatic object detection and astrometric reduction mechanism and a constraint-based filtering of outliers for the final generation of the tracklet. The method was tested on multiple observation sessions for surveying the CLUSTER II highly eccentric orbit satellites, including the CLUSTER II FM5 satellite (Rumba) on its final passes before reentry, and the accuracy of the measurements was estimated based on ground truth from ESA’s reentry team. The method was also tested on lower orbit objects and found to be accurate for objects with ranges of more than 1300 km from the observer.

## 1. Introduction

Precise object tracking of space objects is an image acquisition method that uses the mount of the telescope to orient the instrument in real time towards the target to be tracked, thus compensating for the target’s motion. This method assumes that the orbital parameters of the object are known with a reasonable accuracy. If this assumption is met, the object will appear as a circular or point-like shape in the acquired image, while the background stars will appear as streaks. This method can be contrasted to sidereal tracking, which implies the use of the telescope mount to compensate for the Earth’s rotation, and therefore the stars appear as point-like sources and the moving targets appear as streaks. While sidereal tracking allows us to observe objects even when their orbital elements are unknown or out of date, it will not work for very faint targets. In sidereal tracking, the light reflected by the faint objects is spread over multiple pixels during exposure, while in precise object tracking the light accumulates in the same spot.

**Figure 1:**
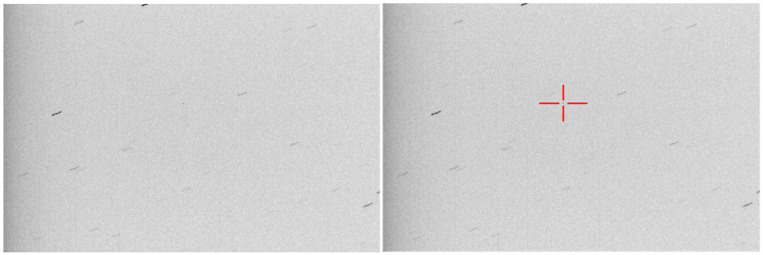
Example of input image. (**Left**)—original acquired image, with an exposure time of 10 s. The target object is barely visible. (**Right**)—the target object is highlighted by a red cross.

Extremely faint objects (small objects or objects at a large distance) can be difficult to observe even in precise object tracking mode. This is the case shown in Figure 1, where the object (a satellite on a high eccentricity orbit) is more than 100,000 km away from Earth. Even at a 10 s exposure time, the object is still faint and does not stand out from the noise or the background stars. An increased exposure time will accumulate more light from the object, but will also cause longer star streaks, which will merge with one another and make the astrometric calibration impossible.

The purpose of the work described in this paper was to design a method to automatically detect objects in sequences acquired in precise tracking mode, focused on faint objects, fast processing time, and minimum user intervention. Therefore, we have focused our effort on increasing the target object’s contrast, on facilitating reliable automatic astrometric calibration for every frame in the sequence, and on automatic rejection of outliers. The theoretical contributions of this paper are a new weighted stacking method for contrast enhancement, the automatic creation of a star filter for creating local maxima for astrometric calibration, and the automatic rejection of false positives by polynomial fitting. The main technological contribution is the creation of a complete image processing pipeline able to quickly generate reliable measurements from image sequences with minimum human intervention.

## 2. Related Work

Active satellites and space debris in all orbits need to be permanently monitored so that their orbital parameters are updated, so that their position can be accurately predicted. The most popular approach to detect these objects from optical images is to use the sidereal tracking mode, using the telescope’s mount to adjust for the rotation of the Earth. This way, the object’s motion will cause its image to appear as a streak and the background stars as point-like sources. This approach is convenient for two reasons: the streak-like shape in the image is an outlier that can be detected with specialized algorithms, and the stars’ point-like shape makes astrometric calibration easier. A detailed description of a method for detecting streaks and stars is presented by Levesque [1]. The motion of the object to be observed is assumed to be known; therefore, a matched filter of specific orientation, length and width can be designed, and then applied by the convolution on the image, in the Fourier domain. The method presents techniques for background modeling and for artefact and star detection and removal, removing everything that is not a potential streak before applying the convolution. A study by Virtanen [2] presents an automated pipeline for streak detection based on a single image. The image is subjected to automated thresholding, and then the non-streak objects are removed by mathematical morphology. A pre-classification of the streak objects is performed by point density evaluation using variable size windows—if the window contains a streak, the number of points in the window should grow linearly with the window size. The final classification is performed by Principal Components Analysis (PCA).

The distinct shape of the satellite streak has inspired recent researchers to use machine learning techniques to detect and classify them. The paper by Calvi [3] describes a system for the detection and classification of objects of interest from a telescope image based on the convolutional neural network YOLO (You Only Look Once). The researchers use real and synthetic data for network training while also applying augmentation techniques. The system was tested for satellites in the LEO (Low Earth Orbit) and MEO (Medium Earth Orbit) regions. Another solution, described in [4], uses semantic segmentation based on the neural network U-Net, which assigns a class to every pixel of the image, to identify large streaks in narrow-field-of-view images. The streak detection is completed by applying the Hough transform to fit a line segment to the streak pixels identified by semantic segmentation.

The use of object tracking, where the target object is a point instead of a streak, became popular when researchers started focusing on space debris at a large distance from the Earth’s surface, such as the space debris in the geosynchronous Earth orbit (GEO), and other highly elliptical orbits, such as the geosynchronous transfer orbit (GTO). The study by Schildknecht [5] presents the campaigns of optical observations performed by the European Space Agency (ESA) in the GEO and GTO regions. The objects in these orbits are far away from the Earth’s surface, making radar observations inefficient and making optical telescopes the instruments of choice. However, the large distance means that the objects are also faint and require prolonged integration of their reflected light. Observing these objects in object tracking mode is simplified by their quasi-stationary position with respect to the Earth.

A comprehensive survey by Schildknecht [6] presents the main issues of detecting and tracking space debris, highlighting the similarity between searching for faint debris and searching for near-Earth objects (NEOs). For the geosynchronous Earth orbit (GEO), the faint debris can be observed by the integration of the light over a longer period of time (several seconds) while the telescope is fixed with respect to the Earth (“parked” position). The exposure time will cause the background stars to be seen as streaks, while the GEO object will be seen as a round shape. The solution proposed for astrometry is to combine sidereal frames, which will show the stars as points, with staring frames for object detection. The observation mode switches between tracking (observing the object) and repositioning for calibration.

The study by Sun [7] highlights the fact that radar and laser observations are useless in the GEO region, and proposes the use of a wider FoV telescope with a 5-s exposure for each frame and 10 consecutive frames for every observation. The star streaks are eliminated by mathematical morphology operations, and then the frames are combined by median filtering for each pixel, to increase the signal-to-noise ratio of stationary targets. The objects are then detected using a morphological operator based on the a priori knowledge of the desired object shape.

Another approach to solve the problem of stars as streaks, which appears when the telescope is either locked in to observe the GEO or is set to follow the object based on its orbital parameters, is to use deconvolution methods, as shown in [8]. The technique involves iterative application of the Adaptive Wavelet Decomposition Maximum Likelihood Estimation (AWDMLE) with a known Point Spread Function (PSF). The technique can work with any PSF (streak-like or circular), and is able to increase the signal-to-noise ratio (SNR) and highlight faint stars and objects. The main disadvantage is that the process is slow, involving hundreds of iterations for a single image. Similarly, Kouprianov [9] presents a method for image processing for GEO and close-to-GEO object detection, solving the problem of trailed (streak) stars by PSF fitting using non-linear least squares. The same paper presents the Apex II software implementing the processing pipeline. A similar method using iterative convolutions with a streak shape to identify stars in object tracking sequences, and methods for bright star detection to identify the tracked object, were initially presented in [10], and then developed into a generic image reduction software product, presented in [11].

A recent paper by Wang [12] presents a new approach for discriminating between the tracked object and the background stars, a method that can be used when the object is a streak and the stars are point-like, and also when the object is a point-like (or round) source and the stars are streaks. The method is based on detecting all objects that have intensities above the background, and then computes a feature vector for each object and classifies objects based on their similarity with the others using the Mahalanobis metric. The outliers are considered objects of interest and the inliers are considered part of the star background.

The method described by Yanagisawa [13], also focused on objects in the GEO region, is based on the process of combining multiple consecutive images, which is called stacking. The stacking process is based on the median operator for pixels in the same position in the image, in consecutive images. The median operator is robust against outliers, and is not strongly influenced by moving background stars. The median is applied to 10 frames, and groups of 10 stacked frames are then combined by averaging, to further increase the signal-to-noise ratio.

Recently, the image sequence reduction solutions are oriented towards generic, client–server, high-processing-power solutions, such as Gendared [11] and CPOSD [14], which are designed to process in a centralized manner the images acquired from heterogeneous sources and perform reductions for all tracking approaches.

Motivated by the participation in an observation campaign for the reentry of a space object of a highly eccentric orbit, posing challenges such as faint magnitude (due to the object’s distance), imperfect modeling of the object’s motion (due to decaying orbit), and the need for fast data processing and delivery, our method is designed to process image sequences acquired in (quasi-) precise object tracking mode without the need for prior knowledge about the star streaks (such as used in [8,9,10]), and without prior astrometric calibration frames with point-like stars (like the methods described in [6]). The method is lightweight and capable of on-site processing, unlike the centralized methods shown in [11,14]. The median stacking process for increasing the target’s contrast is similar to the one shown in [13], but the weighted stacking proposed in this paper is able to improve the detection rate, as we will show in Section 4. The method presented in [12] is focused on detection and discrimination between stars and targets but does not address the problem of contrast improvement or astrometric calibration. To summarize, the proposed method addresses all the problems of quasi-precise object tracking: low contrast, stars as streaks which cause difficulties for calibration, and false-positive rejection, all with minimum human intervention.

## 3. Materials and Methods

### 3.1. Method Overview

We propose a lightweight, fast solution to process image sequences acquired in object tracking mode, using any kind of telescope setup and targeting objects at altitudes from 1000 km to 120,000 km and beyond. The image processing solution does not need prior data about the target or the sky region surveyed, but only a rough estimation of the pixel angular resolution for speeding up the astrometric calibration. Based on the image sequence, the algorithm will generate a Tracking Data Message (TDM) [15] file containing equatorial coordinates and timestamps of the target’s trajectory.

**Figure 2:**
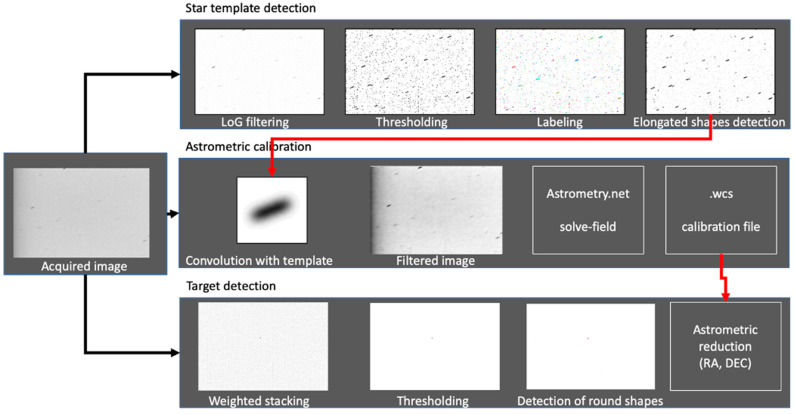
Method overview.

The algorithm has three main parts, which are shown in Figure 2. First, a star template is extracted from the acquired image by a sequence of image processing steps: thresholding, connected components labeling, and extraction of elongated objects. Due to the fact that the star streaks are generated by the telescope’s motion and the field of view is narrow, we can assume that all of the stars generate streaks of the same size and orientation, and therefore we can compute a median of the streaks and use it as our representative star streak shape.

The second part of the solution is the astrometric calibration. Here we use a publicly available tool, Astrometry.net’s *solve-field* [16]. However, this tool is designed for point-like stars, and, as we will show in the Results section, does not work properly with streak stars. By using the star streak template that we have extracted, and filtering the acquired image with it, we will produce an image that will have local maxima in the middle of the star streaks and will work perfectly with *solve-field*.

The third part of the solution is the detection of the target object. The main challenge is to increase the target’s contrast with respect to the background stars and noise. We have achieved this by stacking *K* consecutive frames while giving more weight to pixels of stable value. This approach is similar to Yanagisawa’s [13], which also uses the median for stacking, but instead of using the median, we add the intensity of the pixels weighted by a Gaussian function based on their distance to the median. After increasing the signal-to-noise ratio, we apply automatic thresholding and select the connected objects with low eccentricity.

A post-processing step removes the false positives by filtering the results against a polynomial curve. The filtered equatorial coordinates are then written in the TDM file.

### 3.2. Detection of Star Streaks

**Figure 3:**
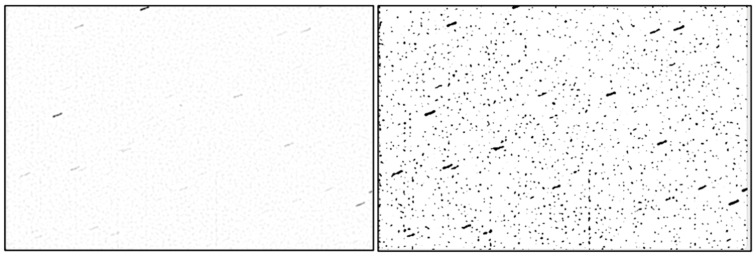
LoG filtering (**left**) and thresholding (**right**) to select the brighter objects in the image.

First, we apply a Laplacian of Gaussian (LoG) filter, with the standard deviation $\sigma_{LoG}$, to remove the uneven background light. The choice of the numerical value $\sigma_{LoG}$ will be explained in the experimental results section (Section 4). The result of applying the LoG filter is shown in Figure 3 (left). Then, the LoG-filtered image is thresholded using an adaptive threshold that selects the brightest 5% of the pixels in the image. The result is shown in Figure 3 (right).

**Figure 4:**
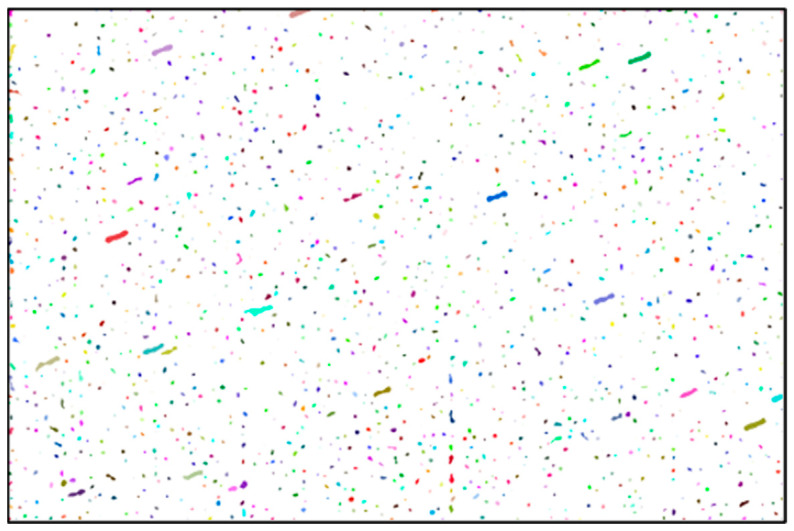
Labeling of connected components.

Next, we apply the algorithm for the labeling of connected components to identify individual objects from the binary image. The result of this operation is shown in Figure 4.

The next step is to identify the objects that can be candidates for star streaks. For a given label image *L*(*x*,*y*) and a label *l*, we compute the central moments of the second order, μ*_l_*_,2,0_, μ*_l_*_,0,2_, and μ*_l_*_,1,1_, using the following equation:

(1) $$\mu_{l,p,q}=\frac{\sum_{x,y}\left[L\left(x,y\right)=l\right]{\left(x-\bar{x_{l}}\right)}^{p}{\left(y-\bar{y_{l}}\right)}^{q}}{A_{l}}.$$

A*_l_* is the area (the number of pixels) of the object of label *l*, and $\bar{x_{l}}$ and $\bar{y_{l}}$ are the coordinates of the object’s center of mass. Based on the second-degree moments, we can compute the orientation angle of the object with respect to the horizontal image axis, *θ_l_*:

(2) $$\tan\left(2\theta_{l}\right)=\frac{2\mu_{l,1,1}}{\mu_{l,2,0}-\mu_{l,0,2}}.$$

Using the same moments, we will then compute the length of the major axis, *D_l_*, and the length of the minor axis, *d_l_*, of the ellipse that best contains the object of label *l*:

(3) $$C_{l}=\sqrt{{\left(\mu_{l,2,0}-\mu_{l,0,2}\right)}^{2}+{\mu_{l,1,1}}^{2}}$$

(4) $$d_{l}=\sqrt{8\left(\mu_{l,2,0}+\mu_{l,0,2}\right)-C_{l}}$$

(5) $$D_{l}=\sqrt{8\left(\mu_{l,2,0}+\mu_{l,0,2}\right)+C_{l}}$$

Based on the length of the two axes, we can compute the eccentricity, *e_l_*, of the object:

(6) $$e_{l}=\sqrt{1-{\left(\frac{d_{l}}{D_{l}}\right)}^{2}}.$$

**Figure 5:**
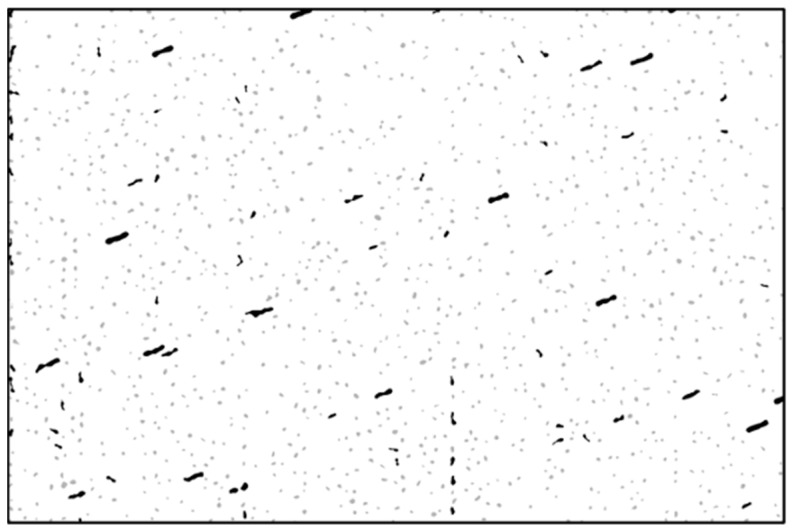
The elongated objects, streak candidates, are depicted in black, while the rest of the objects are depicted in gray.

The objects with high eccentricity and a length *D_l_* above a (small) threshold are considered star streaks. The chosen eccentricity threshold is 0.65, which denotes a moderately elongated object. A higher eccentricity threshold can be set for longer exposures which cause the star streaks to become almost line segments. The purpose of eccentricity thresholding is not to accurately select the stars, but to have a high probability that the chosen objects are the stars. The star streaks are indicated as black objects in Figure 5. The rest of the objects, which have not passed the eccentricity and the length test, are shown in light gray.

Based on the extracted star candidates, we will compute a generic star template using the following steps:

1. Compute the median orientation, median minor axis length and median major axis length for all the objects that are considered likely star candidates.
2. Using the median values, create a star template image:
  - Draw a line of 1 pixel width, of a length given by the median major axis length, and of an orientation given by the median orientation of the streak objects.
  - Convolve the resulted binary image with a Gaussian filter of standard deviation equal to the median minor axis length.
  - Normalize the resulted image such that it can be used as a filter with the sum of the elements equal to 1.

**Figure 6:**
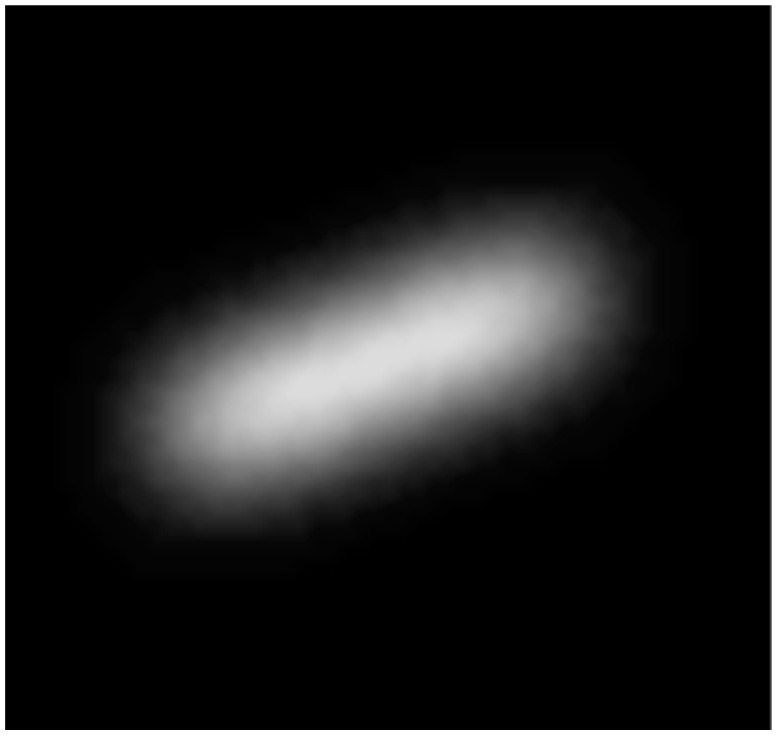
The star template filter computed from the median star streaks.

The filter resulting from processing the streaks from Figure 5 is shown in Figure 6.

### 3.3. Astrometric Calibration

Based on the results of the star detection algorithm, we can use the computed star template to prepare the acquired grayscale images for astrometric calibration. The tools provided by *astrometry.net* are free to use on any Unix-like OS, are capable of performing blind astrometric calibration at any pixel scale if the necessary catalog files are downloaded, and can be quite efficient in processing time if the correct pixel scale is provided a priori. However, these tools expect that the input images contain point-like stars, not streaks. In order to use precise object tracking images with *astrometry.net*’s *solve-field* tool, we have two options:

1. Use the detected star streaks, compute their centroids and generate an *.axy* coordinate file compatible with *solve-field*;
2. Modify the original grayscale image in such a way that the centers of the star streaks will appear as local maxima and let *solve-field* extract its own sources.

**Figure 7:**
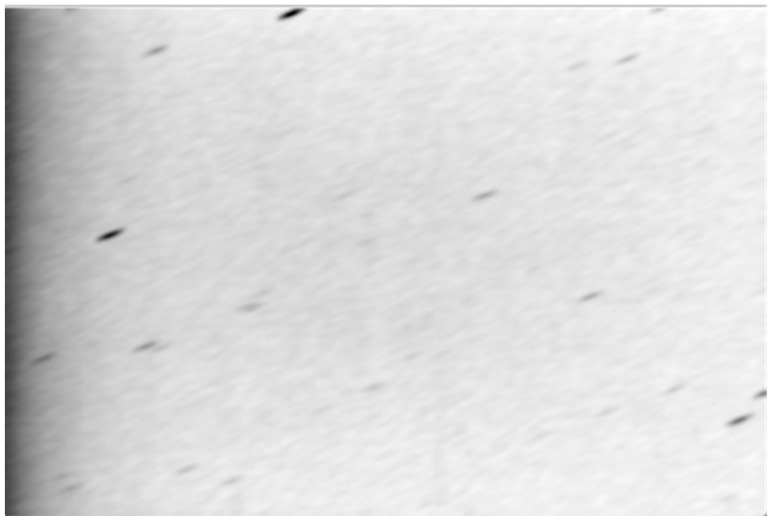
Result of filtering with the star streak template, input for *solve-field*. Negative of the intensity image.

The second option was found to produce the best results. The star streak template, used as a convolution filter, will emphasize the stars and cause local intensity maxima in the middle of the star streaks. As a bonus, convolving the image with such a large filter will also suppress much of the noise, reducing the false source candidates extracted by *solve-field*. The result of the filtering with the star template is shown in Figure 7.

### 3.4. Target Detection

The first step in target detection is to increase the signal-to-noise ratio of the target. We will combine *K* successive frames (LoG-filtered for background removal), using an algorithm that will ensure that stable pixel intensities are favored more than strong but changing intensities. The reason behind this approach is that, by using precise object tracking, the target will have an approximatively stable position, while the stars will move across the image. Thus, given the most recent *K* acquired images, we will first compute an image of the median values of each pixel across the interval:

(7) $$M\left(x,y\right)=median\{I_{1}\left(x,y\right),I_{2}\left(x,y\right),\ldots ,\;I_{K}\left(x,y\right)\}$$

Using the median image, we can now produce the combined (stacked) image by adding the intensity of each pixel weighted by a Gaussian function, which will penalize the difference between the pixel’s intensity and the median:

(8) $$S\left(x,y\right)=\sum_{i=1}^{K}I_{i}(x,y)e^{-\frac{{\left(I_{i}\left(x,y\right)-M(x,y)\right)}^{2}}{{\sigma_{weight}}^{2}}}$$

The choice of *K* depends on how well the known orbital parameters of the target predict its actual motion. If the prediction is accurate, the position of the object remains stable, and a larger *K* will result in a better signal-to-noise ratio for the target, without any negative effect. However, if the object’s position in the image is changing, a large *K* will be detrimental, as the accumulated pixels will not belong to the target object. From our own experiments we have found that a *K* = 5 works well with a slowly moving object, such as the one shown in Figure 1, but if the orbital parameters are unreliable the *K* factor must be decreased to 3, or even to 2 or 1 (*K* = 1 means that we do not perform stacking, but rely on a single image for further processing). An analysis about the influence of *K* and $\sigma_{weight}$ on the detection performance is presented in the Results Section (Section 4).

**Figure 8:**
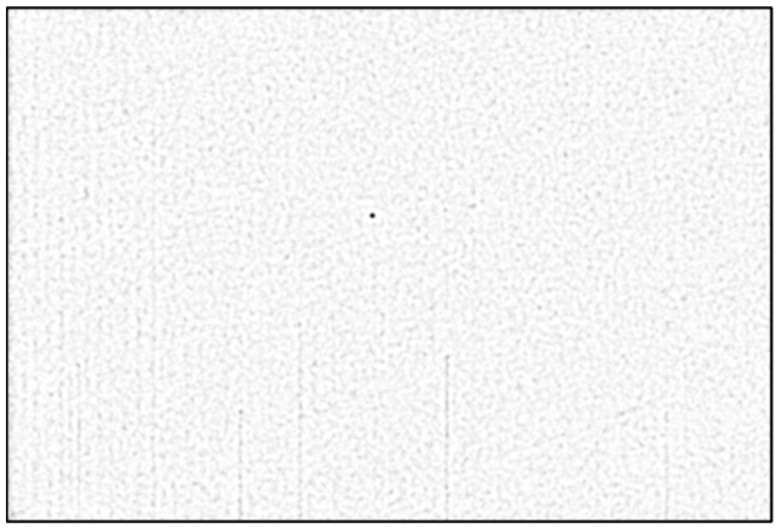
Result of K = 5 image stacking for increasing the target’s signal-to-noise ratio. Negative of the intensity image.

The result of stacking for the object in Figure 1 is shown in Figure 8.

The next steps are similar to the ones performed for star streak detection:

1. Adaptive thresholding, selecting the brightest 1% of the pixels in the stacked image;
2. Connected components labeling;
3. Computation of the object size and eccentricity using Equations (1)–(6);
4. Selection of the objects of low eccentricity (circular objects), with a major axis higher than a moderate threshold, just to exclude point-like noisy objects (ideally this threshold should not be necessary, but hot pixels or other types of noise can cause small noise objects to appear). The eccentricity threshold for the target detection process is 0.60, because a target that will not be stationary in the image space will not be perfectly round in the stacked image.

**Figure 9:**
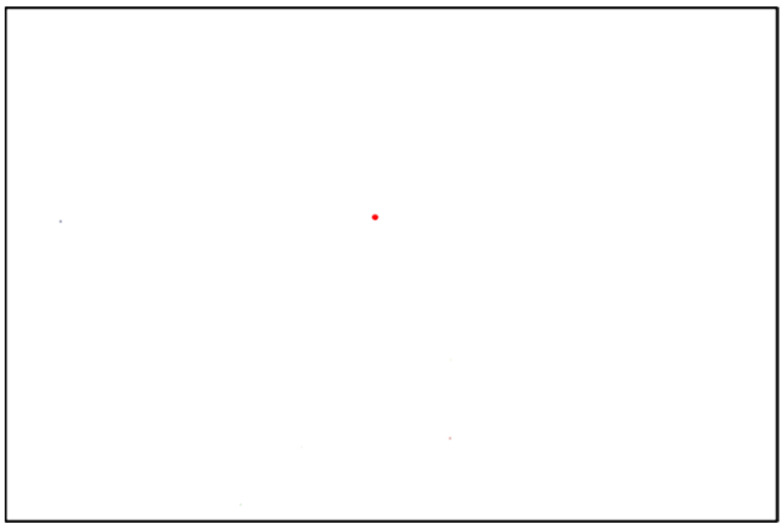
A successfully detected object.

A successfully detected object is shown in Figure 9.

The image space coordinates for the current frame *F* are paired with the astrometric results of a previous frame *F*-*K*/2 (*K* being the number of stacked images), if available. The reason for this approach is that, even though the telescope follows the object, it may not match its motion exactly, and the position of the object may change in the image space. By combining *K* frames and assuming a constant apparent motion of the object, the center of mass of the result will be the position of the object in the frame *F*-*K*/2.

Using the calibration results, the coordinates of the center of mass of the detected object are converted to equatorial Right Ascension and Declination coordinates using the *wcs-xy2rd* tool from *astrometry.net*. Thus, the results of processing each frame are pairs of (RA and DEC) equatorial coordinates for each detected object. If calibration results are not available for the *F*-*K*/2 current frame, the detection results are discarded. Due to the fact that the background stars are moving, we cannot use calibration results of other frames.

An accuracy comparison between the use of the *F*-*K*/2 calibration results and the use of the latest calibration results (for frame *F*) will be shown in the Section 4.

### 3.5. Tracklet Formation

The final step is to form the tracklet and generate the TDM file. The main challenge at this step is the presence of false positives that may remain in the set of detected points, especially when the target is faint, and we cannot impose very strict shape and size constraints on the detection algorithm. We can, however, impose a continuity constraint on the trajectory of the target, expressed in equatorial coordinates. Even if, during the observation time, the position of the telescope undergoes several adjustments, leading to abrupt changes in the target position in the image, the equatorial coordinates should define a continuous trajectory in time. We used this property to select the final set of points to define our tracklet.

The point selection algorithm is based on the RANSAC (RAndom SAmple Consensus) method. We will assume that the points must lie on a polynomial curve of a set degree *N*. The steps of the algorithm are the following:

1. Computation of the *N*-degree polynomial trajectory:
  - Select a random set of *N* points;
  - Fit polynomials of degree *N* to the pairs (time and RA) and (time and DEC);
  - Compute the number of inliers (the points that are closer to the fit polynomials by a distance threshold);
  - Keep the polynomial parameters if the number of inliers is maximum;
  - Repeat steps a. to d. 10,000 times;
  - Return the polynomials that produce the maximum number of inliers.
2. Selection of the final set of points for the tracklet:
  - For each timestamp of the detection points, compute the value of RA and DEC given by the best polynomial model;
  - Compute the distance between (RA and DEC) of the point and (RA and DEC) predicted by the polynomial;
  - If the angular distance is below a threshold, the point is considered inlier and part of the tracklet.

**Figure 10:**
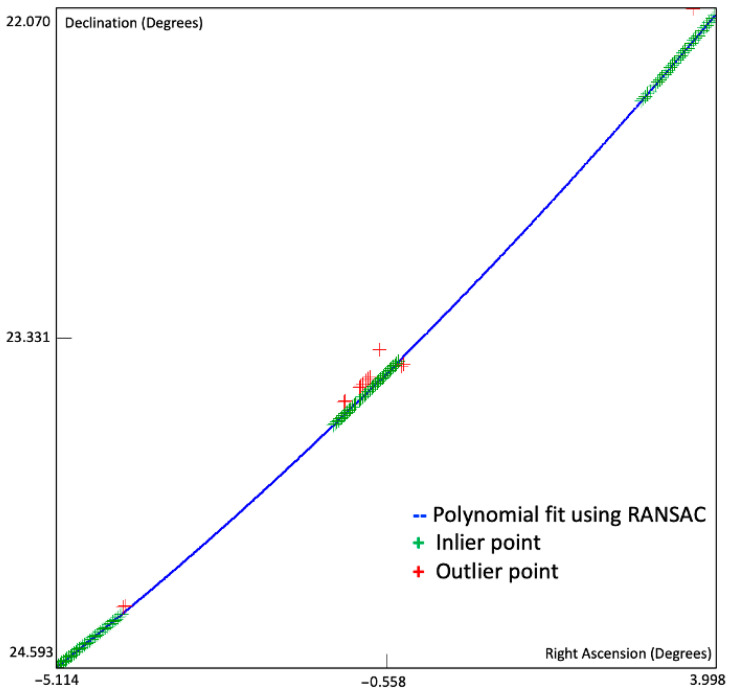
Filtering the detection points for tracklet formation: the inlier points, close to the curve, are accepted, while the outliers are rejected.

**Figure 11:**
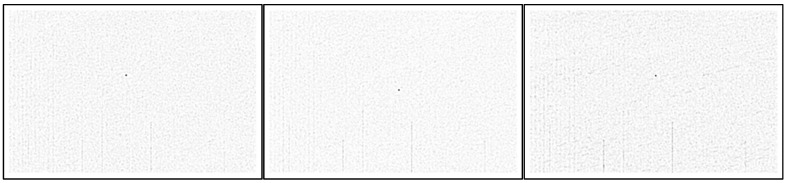
Repositioning of the telescope for three continuous sequences affects the position of the target in the image space but will not affect the final result.

The process of point selection is depicted in Figure 10. The blue line depicts the best polynomial curve, the green points are the inliers that are considered part of the tracklet, and the red points are the outliers to be rejected. The sequence was acquired during a 1 h 30 min observation campaign containing three 8-min continuous sequences separated by breaks of approximatively 30 min. During the breaks the telescope was repositioned and the position of the target in the image changed, as shown in Figure 11. The repositioning did not affect the measured trajectory of the object, expressed in equatorial coordinates.

## 4. Tests and Results

### 4.1. Calibration Performance and Execution Time

The first test compares the calibration performance of *Astrometry.net*’s *solve-field* with and without star streak pattern filtering. To speed up the performance, in both scenarios we restrict the pixel scale values to be close to the actual value, but no other restrictions are applied (no guessing of the initial plate center). If the pixel scale is not known (a new sequence from a new instrument), we can call *solve-field* without any restrictions, and use the initial calibration results for the pixel scale interval. The calibration time is restricted to 60 s—if no solution is found in 1 min, the calibration fails.

For a sequence of 40 images, we compared the calibration success rate and the average frame time. The results are shown in Table 1.

**Figure 12:**
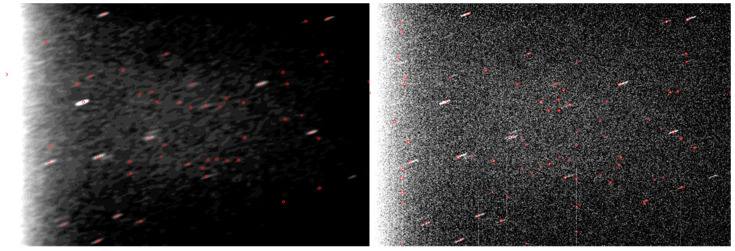
Solve-field extracted sources: (**left**): from the streak template filtered image; (**right**): from the original image.

The results clearly show the advantage of using a star streak pattern filter to improve the calibration performance, both in terms of the success rate and time performance. The explanation for this improvement can be partially explained by the comparison shown in Figure 12. The sources extracted by *solve-field* are fewer and more accurately positioned in the filtered image compared to the original image.

The execution time depends mostly on the astrometric calibration time. The processing steps described in this paper take less than 1 s for a FITS image of size 1536 × 1024 pixels on a 2020 MacBook Pro M1 laptop. The average time is less than 10 s for a frame, meaning that the sequence can be processed in less time than was required for its acquisition, or it can be processed in real time.

### 4.2. The CLUSTER II Observation Campaign and Rumba’s Reentry

Taking advantage of the successful experience with the controlled reentry of the Salsa satellite (CLUSTER II-FM7, NORAD ID 26411, COSPAR ID 2000-041B) which took place on 8 September 2024, the ESA invited us to participate to the optical observation campaign for the Rumba satellite (CLUSTER II-FM5, NORAD ID 26463 COSPAR ID 2000-045A), which was also planned for a controlled reentry for the end of October 2025 [17,18]. The reentry campaign of optical observations for the Salsa satellite demonstrated the feasibility of using multi-sensors optical only observations for accurate orbit determination (OD) and reentry prediction as a back-up plan for the future reentries of the remaining CLUSTER-II satellites [19].

During 12/13 May–20/21 October 2025, we performed 25 observation sessions for the reentry campaign for Rumba, including additional observations in 17 observing sessions for the Samba and Tango satellites. The observations were performed from the Feleacu Station of the Astronomical Observatory Cluj-Napoca (GPS coordinates: Latitude: 46.710073° N; Longitude: 23.593564° E; Altitude: 783.4 m) using a PlaneWave CDK24 telescope (PlaneWave Instruments, 1375 North Main St., Bldg. #1 Adrian, MI 49221, USA, https://planewave.com/) with a focal length of 3968 mm and an aperture diameter of 610 mm. The telescope was mounted on a PlaneWave L600 DirectDrive equatorial mount (PlaneWave Instruments, 1375 North Main St., Bldg. #1 Adrian, MI 49221, USA, https://planewave.com/), capable of a slew speed of up to 50 degrees per second, allowing us to track even fast-moving Low Earth Orbit satellites. An astronomical CCD camera (SBIG STL-6303E, by Diffraction Limited, 5-33 Roydon Place, Ottawa, ON K2E 1A3, Canada, https://diffractionlimited.com/legacy-product-support/, accessed on 1 February 2026) was placed in the focal plane of the telescope, and the acquired images were non-filtered 16-bit monochrome FITS, with 1536 × 1024 pixels in 2 × 2 binning mode. The pixel scale was determined to be 0.93 arc seconds/pixel, and the field of view size was 23.8761 × 15.9286 arcmin. The exposure time was varied between 5 and 20 s, depending on the angular speed of the target (a shorter exposure for faster objects and a longer exposure for slower objects which were further away). Taking into account the Rumba satellite size and its extremely eccentric HEO orbit (eccentricity > 0.83), with an apogee distance > 131,000 km, we used object tracking mode as the main procedure to obtain observations.

The mount has a satellite tracking mode which uses satellite Two-Line Elements (TLEs) as input in the telescope control software PW4 (software Plane Wave Interface 4 (PWI4), version 4.1.6, https://planewave.com/software-updates, accessed on 1 February 2026). Until the end of September 2025, we used publicly available TLEs from *space-track.org* for Rumba. For the early October 2025 observation sessions we used TLEs calculated in the orbit determination (OD) process by the ESA Flight Dynamics (FD) team due to the lack of publicly available accurate TLEs.

On 16 June 2025, we observed all three CLUSTER II satellites for an extended period of time. Each object was tracked in three continuous sub-sequences. The images were then processed automatically, and the TDM files were generated. Since we had no accurate ground truth data, we could only compare the results to predictions generated by the Orekit library based on the latest available orbital parameters (TLE format) downloaded from *space-track.org*. The computed errors are shown in Table 2. While the errors are considerably higher than the expected arc second accuracy, they can be explained by the limitation of the orbital parameters and the limitation of the prediction model. A possible explanation is that Orekit does not take into consideration the light travel time from the satellite to the observer, and this time is significant for such distant satellites.

**Figure 13:**
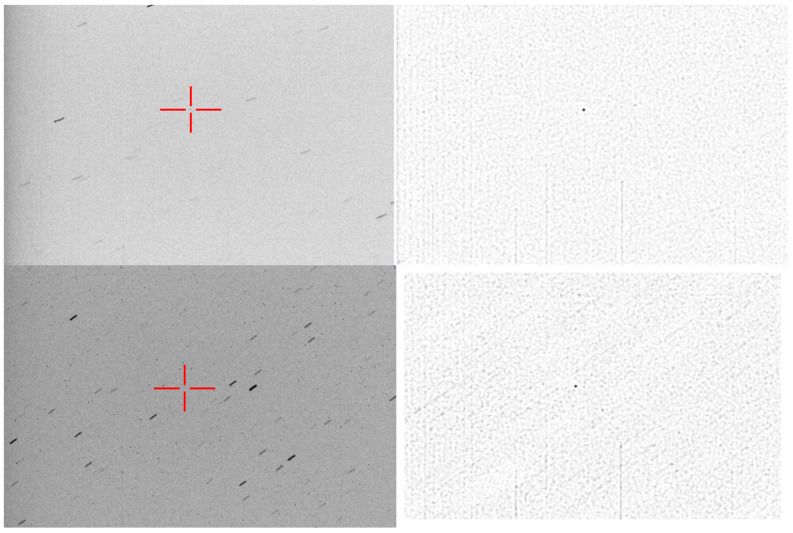

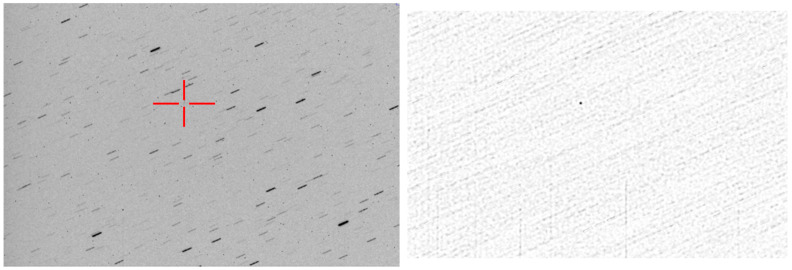
Comparison between negative single frame and stacking ((**top**)—Rumba, (**middle**)—Samba, and (**bottom**)—Tango).

The comparison between the single frame aspect and the stacked aspect of the Cluster II tracked targets is shown in Figure 13. We can see that, while the closer target, Rumba, can be observed in the single image, it is almost impossible to single out the targets that are farther away, Samba and Tango, without the use of stacking.

**Figure 14:**
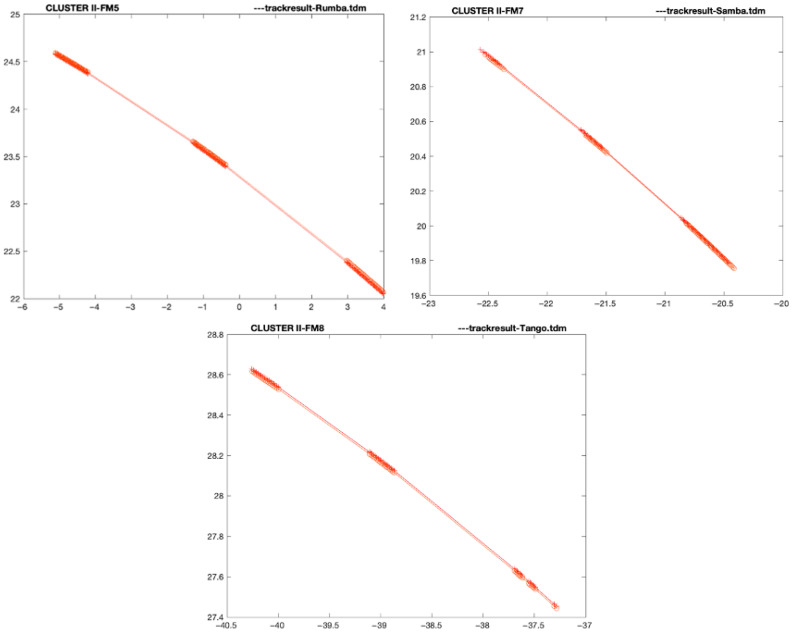
Comparison between predicted and observed trajectories for the Cluster II satellites Rumba, Samba and Tango. The horizontal coordinates are Right Ascensions (in degrees) and the vertical coordinates are Declinations (in degrees).

The purpose of the preliminary experiment is only to test the detection performance and the robustness against false positives. The comparisons between the predicted and the observed trajectories for the three Cluster II satellites is shown in Figure 14. The predicted positions from the known orbital parameters, using the Orekit library, are shown by the ‘+’ symbol, and the observed positions are shown by ‘o’. The horizontal axis shows the Right Ascension coordinate and the vertical axis shows the Declination, both in angular degrees. Even though the trajectories are not perfectly overlapped, the measured curve closely follows the prediction, with a constant offset, which can be explained by inaccurate orbital elements or a limited prediction model.

For accurate validation of the measurement accuracy, we used the sequences acquired in August and September 2025, part of the Rumba reentry observation campaign. The TDM file datasets, generated based on our observations, were analyzed, validated, used and acknowledged by the ESA’s Rumba reentry team. The accuracy results for the August and September sessions are shown in Table 3.

**Figure 15:**
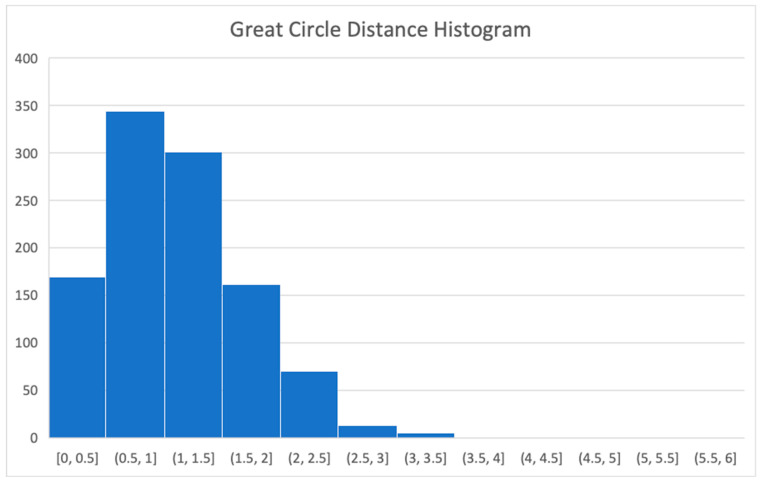
Histogram of the great circle angular distance values for all measurements in August and September 2025.

A histogram of the great circle angular distance values between all measurement points obtained in the August–September campaign and their corresponding ground truth values provided by the ESA reentry team is shown in Figure 15. We can see that most errors are within the interval 0.5–1.5 arc seconds, with very few outliers.

On the last observing session before Rumba’s reentry, during the night of 20/21 October, the satellite was just after the last perigee passage and the uncertainty for the estimate of the drag coefficient Cd played a major role in the calculated ephemeris, and consequently in the pointing and tracking of the telescope. This meant that the telescope was not able to accurately follow the satellite’s trajectory and the target’s position in the image plane was not fixed. However, a significant amount of overlap between consecutive frames allowed the stacking and target detection mechanism to work.

Again, the TDM file datasets, generated based on our observations, were analyzed, validated, used and acknowledged by the ESA’s Rumba reentry team. The results are shown in Table 4. All observations were performed on the same date, 20 October 2025.

**Figure 16:**
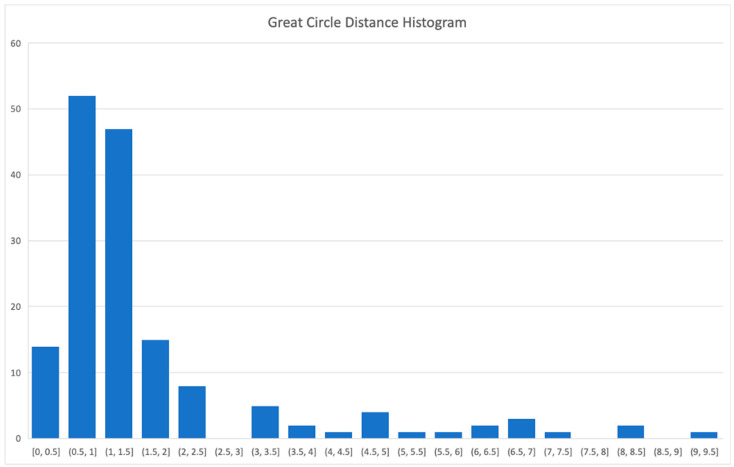
Histogram of the great circle angular distance values for all measurements on 20 October 2025.

A histogram of the great circle angular distance values for all measurement points obtained on 20 October is shown in Figure 16. While most of the errors are still in the interval 0.5–1.5 s, there are some outliers.

The pixel angular size of the telescope and camera system was 0.93 arc seconds/pixel, which means the measurement errors, which include image processing, astrometric calibration, astrometric reduction, timestamp errors, are mostly within the range of 1 pixel.

**Figure 17:**
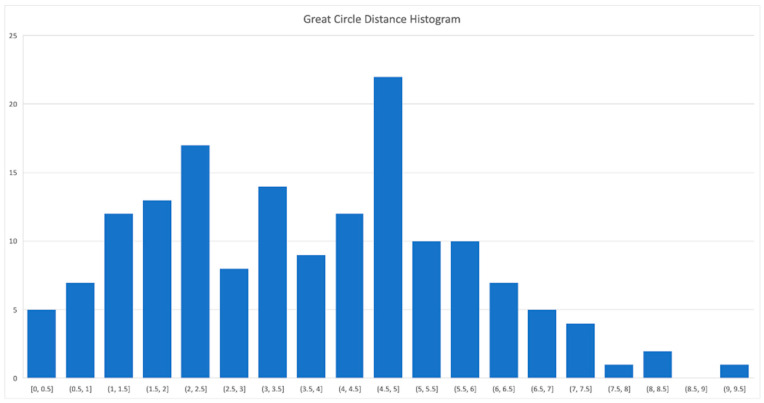
Histogram of the great circle angular distance values for all measurements on 20 October 2025, using the astrometric calibration of the last frame.

The observations before reentry, lacking accurate orbital data for precise orientation of the telescope, show the importance of adjusting the astrometric reduction with the middle of the stacking sequence. If we use the calibration generated by the last frame in the sequence, and the target is not stationary in the image plane, the errors increase, as shown in Figure 17.

### 4.3. Testing the System on Objects of Lower Altitude

While the method presented in this paper was primarily designed for the observation of high-altitude objects, we have also experimented with lower-altitude objects, such as Low Earth Orbit (LEO) objects. Usually, we observe these objects in sidereal tracking mode, detecting them as streaks against point-like background stars. However, if the objects are faint, their reflected light will spread along many pixels in the image and the object will not be observed.

First, we observed the space debris ARIANE 44L DEB (SPELDA) (NORAD ID 20718) on 14 November 2025, and COSMOS 252 DEB (NORAD ID 03555) on 19 December 2025. The Ariane space debris is a larger object (according to satcat.com, it has a length of 2 m and a large radar cross-section of 12 m^2^). The Cosmos 252 debris is a smaller object (according to satcat.com, it has a length of 0.6 m and a medium radar cross-section between 0.1 and 1 m^2^).

On 19 January 2026, we tested the system on three geodetic satellites: EGS (AJISAI)—Experimental Geodetic Satellite (NORAD ID 16908), a reflective sphere of 2.15 m in diameter and 685 kg, orbiting the Earth at an altitude of 1488 km, with a large radar cross-section; LAGEOS 2—Laser Geodynamic Satellite 2 (NORAD ID 22195), a reflective sphere of 60 cm in diameter and 405 kg, orbiting the Earth at an altitude of 5800 km, with a medium radar cross-section; and STARLETTE—Satellite de Taille Adaptee Avec Reflecteurs Laser Pour Les Etudes de la Terre (NORAD ID 7646), a small reflective sphere of 24 cm in diameter and 47 kg, orbiting the Earth at an altitude of 800 km, with a medium-to-small radar cross-section.

The measurements were performed from the Feleacu Station of the Astronomical Observatory Cluj-Napoca, using an Orion ShortTube 80 refractor telescope (Orion Telescope & Binoculars, 93 Stoneybrook Road, Melbourne, FL) with a focal length of 400 mm and an aperture diameter of 80 mm, connected to a SBIG STT-1603 3 ME CCD camera. The image size was 768 × 512 pixels in 2 × 2 binning mode, the pixel scale was 9.26 arc seconds/pixel, and the field of view had a size of 1.96932 × 1.31882 degrees. This telescope and camera setup was mounted on top of the CDK telescope, sharing the same high-performance PlaneWave L600 mount. This setup is normally used for the observation of LEO satellites in sidereal tracking mode. For all objects the exposure time was 1 s.

The computed errors between the measurements and the predicted coordinates generated by Orekit based on the latest available orbital parameters are shown in Table 5.

**Figure 18:**
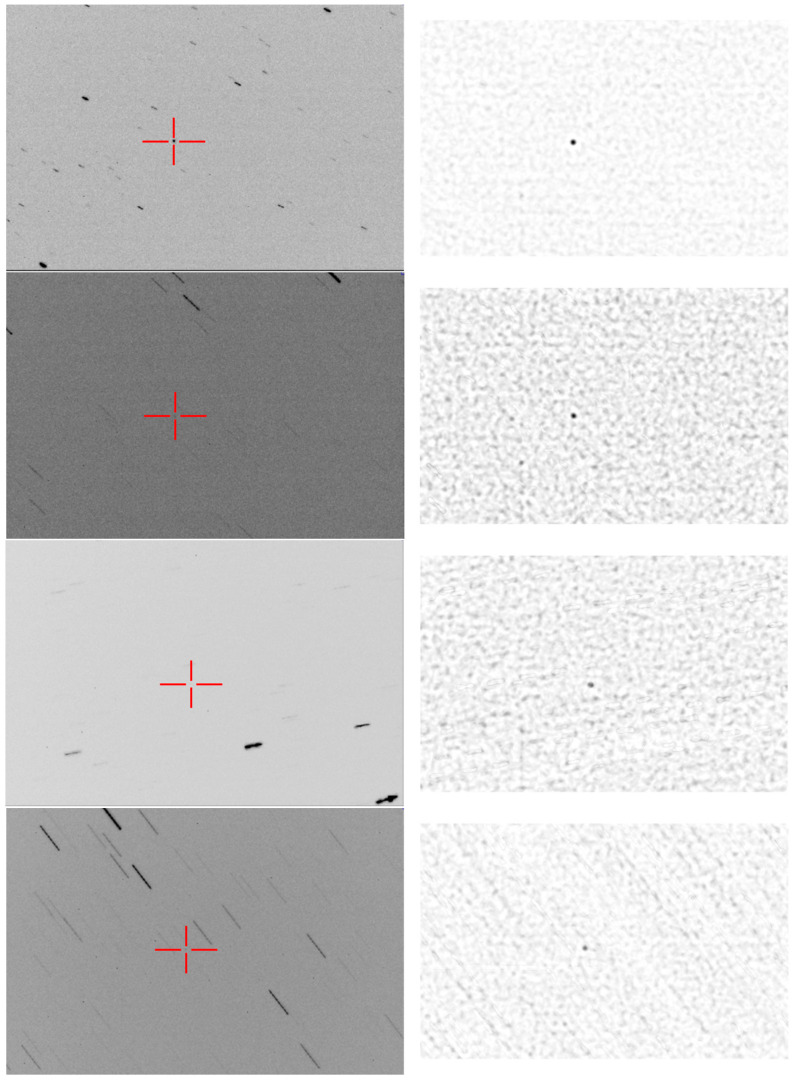

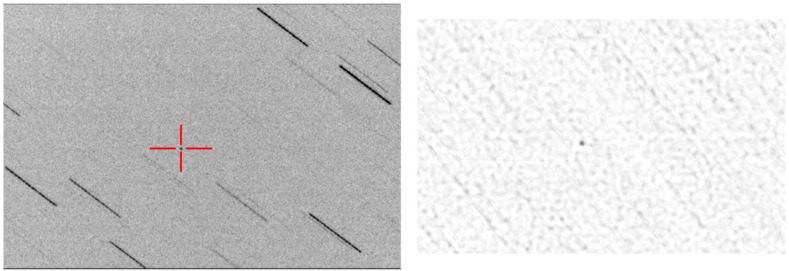
Comparison between single frame and stacking for lower-altitude objects (from top to bottom: ARIANE 44L DEB (SPELDA), COSMOS 252 DEB, EGS (AJISAI), and LAGEOS 2, STARLETTE). (**Left column**): the single frame negative image; (**right column**): stacked image.

The comparison between the single frame aspect and the stacked aspect of the tracked targets is shown in Figure 18. The Ariane space debris is a larger object and is visible in a single frame, and the stacking process is optional, but helps increase the contrast against the background stars and noise. The Cosmos 252 debris is a smaller object, and the single frame does not show it clearly. The EGS and the LAGEOS satellites are difficult to see in the single frame, but the stacking process makes them clearly visible. The STARLETTE satellite is closer to the observation point and easier to spot in the single frame, and can be detected without stacking, but the stacking process increases its SNR against stars and background noise.

The measurement accuracy was estimated based on up-to-date TLE files downloaded from *space-track.org*. The differences between prediction and measurement are shown in Table 4. As expected, the geodetic satellites show the best match between the measurement and the prediction. The best results were obtained for the EGS satellite (sub-pixel measurement errors) and for the LAGEOS 2 satellite (1.5 pixel errors). All of the errors are most likely due to the prediction process, as they show a systematic bias. Even for geodetic satellites with their orbit precisely measured, the difference in prediction while using different orbital parameters (TLE files) can be significantly more than the errors shown in the table (for example, the previous TLE set for LAGEOS 2 showed up to a 100 arc second error with respect to our measurement).

The STARLETTE errors are much larger, and the number of detected points is very low. The limitation is due to the high angular speed of the telescope, which is required to track an object with such a low altitude. The angular speed was 0.33 degrees/second, which caused the stars to produce very long trails in the image, causing superpositions and confusion in the astrometric calibration process. This result shows the limitation of our system: the best results are obtained for objects farther away, beyond the range of 1300 km. However, more experiments should be carried out with lower exposure times.

### 4.4. Comparison with Existing Techniques

In order to validate our proposed method against an existing product, we have used the software tool MPO Canopus (Version 10.8.4.1) from BDW publishing [20]. This tool requires field calibration based on Flat, Bias and Dark frames, fine-tuning of the star streak parameters (length, width, and orientation), and fine-tuning of the parameters of the target (target radius and dead zone radius). The target must be manually selected, and the tool is able to perform astrometric reduction automatically as long as an approximate field center position is indicated, along with an approximate pixel scale. Once all of these requirements are met, MPO Canopus is able to compute the target’s position, both in pixels and in RA/DEC equatorial coordinates, the Full Width at Half Maximum (FWHM), SNR, and magnitude.

Due to the laborious nature of the MPO Canopus manual frame-by-frame reduction, we have limited the comparison to 38 frames from the 2025-08-08 Rumba sequence, 16 frames from the 2025-09-15 Rumba sequence and 20 frames from the 19:54–20:01 sequence and the 2025-10-20 sequence, which shows a non-stationary target due to decaying orbital parameters. Since MPO Canopus computes FWHM, SNR and magnitude, Table 6 also shows the average of these values for the three selected sequences.

While the experiments show that the method proposed in this paper is superior to MPO Canopus, there are still errors which are comparable to the angular size of the pixel and higher than the theoretical FWHM/SNR resolution. The errors between the measurement results and the ground truth positions can result from three possible sources: errors in finding the centroid in the image space (target position in pixels), astrometric calibration errors, or even errors in the ground truth dataset.

As MPO Canopus also provides the image (pixel) coordinates of the target with sub-pixel accuracy, we had the idea of using these coordinates along with our calibration results. The performance improved, as shown in Table 7.

**Figure 19:**
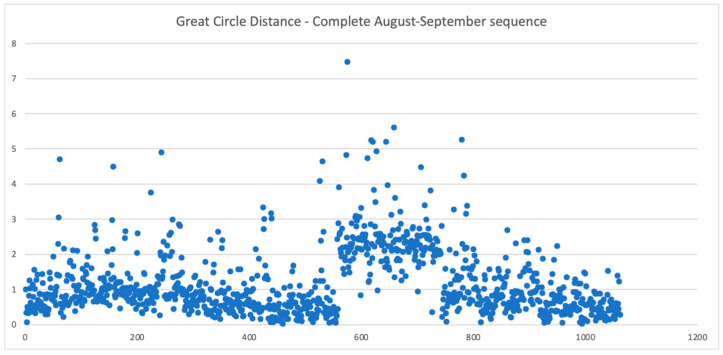
The angular distance between our measurement and the ground truth for the August–September campaign.

From Table 7 we can reach the conclusion that our calibration method is superior, but MPO Canopus’s centroid finding method is better. However, for the 15 September sequence the improvements are not significant. When analyzing the whole August–September dataset (Figure 19), we can see that the great circle errors for frames 561 to 745, corresponding to the 15 September sequence, are clearly outliers. Therefore, we assume that some errors (differences between measurement and ground truth) can also be explained by errors of the ground truth data.

### 4.5. Tuning the Algorithm Parameters

The method presented in this paper relies on several algorithm parameters that can be adjusted by the user. These parameters are

*K*—the number of frames to be stacked;

*N*—the degree of the interpolation polynomial for results filtering;

$\sigma_{LoG}$—the standard deviation for LoG filtering;

$\sigma_{weight}$—the standard deviation for the weighted stacking.

The degree of the polynomial defines the flexibility of the interpolation. For a short sequence, 10–20 frames, a second-degree polynomial should be enough. The majority of our sequences had more than 100 frames, and we have found that *N* = 5 is the best choice. An indication of a good polynomial degree choice is that the polynomial will not mark as outliers continuous groups of points, either at the ends or in the middle, but will only reject true individual outliers.

After choosing the proper polynomial degree, a reliable indicator of the method’s success is the number of inlier measurement points. If we consider as outlier anything that has a distance with respect to the polynomial curve higher than the pixel’s angular size, the number of inliers will indicate the performance of the combination of detection sensitivity and astrometric calibration accuracy.

For selecting the best parameters, we have experimented with three values for K (3, 5, and 7), seven values for the LoG standard deviation (1 to 7), and 10 values for the stacking weighting standard deviation (0.1 to 1). We have chosen two sequences, one where the target is almost stationary in the image space (it moves only by 0.2 pixels between consecutive frames) and one where, due to the decaying nature of the object’s orbit, the telescope does not follow its trajectory accurately and the object’s movement in the image spade is 2.5 pixels/frame. The best parameters for the two sequences are shown in Table 8. As expected, when the target is stationary a higher number of stacked frames and a lower LoG standard deviation produce the best results, but when the target’s position in the image changes fast, a lower *K* and a higher LoG standard deviation are better. The LoG filter size is able to ensure that at least some traces of the previous object’s position overlap with the current frame; therefore, the LoG standard deviation is a measurement of the positional uncertainty of the object.

The best value for the stack weighting standard deviation was 0.8, a higher value causing a gradual decrease in performance. The optimal value was not influenced by the nature of the object’s motion. We believe that this value is dependent on the image noise, and using the same instruments for both sequences caused the noise level to be the same.

While selecting the best parameters leads to optimum performance, the method is not highly sensitive to them. When observing a new object, we can use a set of default parameters: *K* = 5, $\sigma_{LoG}$ = 5, and $\sigma_{weight}$ = 0.8. For these parameters, the 8.08.2025 sequence produces 135 valid measurements and the 21.10.2025 sequence produces 36 valid measurements.

We compared the 8.08.2025 weighted stacking results with the baseline median stacking method described in [13] in terms of valid detection points. The results are shown in Table 9.

### 4.6. Limitations of the Method

The limitations of the method are related to the aspect of the target object and the stars in the image space. These aspects derive from the properties of the target’s motion and from the acquisition process. For example, the star streak’s length can be caused by a long exposure time, but also by the high angular motion of the target. A low SNR can be caused by a small target, by a low sensitivity sensor, or by a small exposure time.

The main limitation parameters are the following:

SNR—The sequence with the smallest SNR that was successfully processed was the 16.06.2025 Samba sequence, with an SNR = 3 for an object magnitude of 17.

Target motion (pixels/frame)—The maximum amount of image space motion, caused by the decaying nature of the object and the impossibility of the orbital parameters to accurately model its motion for a successfully processed sequence, was 2.5 pixels/frame in a sub-sequence from the 20.10.2025 observation campaign.

Star streak length—The maximum star streak length, relative to the image size, was on the 19.01.2026 STARLETTE sequence, which was almost impossible for the astrometric calibration tool. The streak length was 12% of the image width. A long streak length prevents a successful astrometric calibration, even though the target was successfully detected in the image space.

## 5. Conclusions

In this paper we have presented a lightweight solution for the generic processing of images acquired in object tracking mode. This solution has the following characteristics:

- Improves the faint objects’ SNR by weighted stacking;
- Uses the star shape convolution only for star highlighting, not for star detection;
- Uses freely available tools for astrometric calibration;
- Rejects false positives by polynomial trajectory fitting;
- Has real-time performance capabilities: the average processing time, on a medium-power laptop without the use of GPU or other parallel processing capabilities, is less than 10 s/frame, which means that it can be used to process the images as they are acquired.

The method was employed in the surveillance of Cluster II objects, including the observations preliminary to the reentry of CLUSTER II FM5 Rumba, and the accuracy was validated by the ESA’s reentry team. The practical importance of the solution is proven by its fast processing capability of multiple image batches during the reentry campaign in the night after the last perigee pass, and further tests, including LEO surveillance, show that it has a broad applicability. The limitations are imposed by the apparent movement of the satellite in the image space (due to incorrect trajectory modeling), low SNR, or star streaks that are too long in the image. A precise guarantee of the limits of performance for our system is not possible at this point since we have used only two instruments.

Future work will include efforts to improve the measurement accuracy by analyzing and calibrating the sources of errors (image processing, astrometric calibration, telescope mount motion, etc.) and by refining the extraction of the object centroid to increase the sub-pixel accuracy. Since we have found that the centroid detection method used by MPO Canopus is able to significantly improve our results, future work will include further refinement of the object position to achieve better sub-pixel accuracy.

Future work will also include participation in the surveillance of the reentry process of the other CLUSTER II objects.

## Figures and Tables

**Table 1:** Calibration performance and execution time comparison.

	Original Image	Star Streak Pattern Filtered Image
Calibration success	15	39
Calibration failure	25	1
Average frame time (s)	55	6

**Table 2:** Preliminary measurement accuracy results for the CLUSTER II satellites.

Satellite	Number of Valid Measurements	Average RA Difference (Arc Seconds)	Average DEC Difference (Arc Seconds)	Mean Absolute Error RA (Arc Seconds)	Mean Absolute Error DEC (Arc Seconds)	Average Great Circle Angular Distance (Arc Seconds)
CLUSTER II-FM5—range 55,979 km	110	−10.8464	57.2567	14.1959	57.2567	59.3416
CLUSTER II-FM7—range 123,143 km	72	138.7539	−102.0850	138.7539	102.0850	165.4515
CLUSTER II-FM8—range 113,679 km	119	3.2241	−36.8428	3.2241	36.8428	36.9649

**Table 3:** Measurement accuracy results for the CLUSTER II-FM5 (Rumba) satellite, August–September 2025.

Date (D.M.Y)	Number of Valid Measurements	Average RA Difference (Arc Seconds)	Average DEC Difference (Arc Seconds)	Mean Absolute Error RA (Arc Seconds)	Mean Absolute Error DEC (Arc Seconds)	Average Great Circle Angular Distance (Arc Seconds)
08.08.2025	102	0.0094	0.6518	0.6821	1.0087	1.0087
09.08.2025	109	0.2527	0.6005	0.3976	0.7241	0.7241
16.08.2025	112	0.0221	0.6231	0.4403	0.7667	0.7667
29.08.2025	119	−0.4704	0.6300	0.7016	1.0088	1.0088
02.09.2025	116	−0.5557	0.5991	0.8050	1.0733	1.0733
15.09.2025	185	1.5302	0.7406	1.5302	1.7268	1.7268
17.09.2025	55	−0.7217	0.5892	0.7973	1.0837	1.0837
21.09.2025	57	−1.0823	0.6352	1.0823	1.3496	1.3496
22.09.2025	69	−0.4323	0.6146	0.6451	0.9500	0.9500
27.09.2025	138	−0.7115	0.5217	0.7359	0.9491	0.9491

**Table 4:** Measurement accuracy results for the CLUSTER II-FM5 (Rumba) satellite before reentry.

Time Interval (UTC)	Number of Valid Measurements	Average RA Difference (Arc Seconds)	Average DEC Difference (Arc Seconds)	Mean Absolute Error RA (Arc Seconds)	Mean Absolute Error DEC (Arc Seconds)	Average Great Circle Angular Distance (Arc Seconds)
19:54–20:01	20	−0.0856	−0.7034	0.5192	0.7366	0.9690
20:17–20:23	9	0.1804	−0.2110	0.5351	0.5661	0.8219
21:03–21:17	29	0.0966	−0.2496	1.1900	0.6899	1.4401
21:50–22:03	28	0.4602	−0.5398	0.8997	0.7313	1.1860
22:27–22:43	33	−0.8864	−0.3702	1.8375	0.8687	2.0281
23:00–23:22	32	−0.3595	−0.7625	0.7477	0.7912	1.2182

**Table 5:** Measurement accuracy results for lower altitude satellites.

Satellite	Number of Valid Measurements	Average RA Difference (Arc Seconds)	Average DEC Difference (Arc Seconds)	Mean Absolute Error RA (Arc Seconds)	Mean Absolute Error DEC (Arc Seconds)	Average Great Circle Angular Distance (Arc Seconds)
ARIANE 44L DEB (SPELDA)—8418.48 km range	181	27.8736	6.5809	27.8736	6.5809	27.3179
COSMOS 252 DEB—1332 km range	12	30.8265	−13.4837	30.8265	15.8436	33.2818
EGS (AJISAI)—1482 km range	56	1.3547	−5.5051	8.6670	7.7374	12.9205
LAGEOS 2—5734 km range	62	14.4897	14.3234	14.6730	14.5179	20.9389
STARLETTE—1040 km range	3	35.3516	55.0945	35.3516	55.0945	66.2487

**Table 6:** Performance comparison against the tool MPO Canopus.

Sequence	Average SNR	Average FWHM (Pixels)	Average Magnitude	Average Great Circle Angular Distance (Arc Seconds)—MPO Canopus	Average Great Circle Angular Distance (Arc Seconds)—Proposed Method
8.08.2025 (38 frames)	11.30	2.5	15.5903	2.1780	1.0164
15.09.2025 (16 frames)	46.21	2.92	13.91	3.2129	1.6494
20.10.2025, 19:54–20:01 (20 frames)	38.50	2.20	14.78	1.9953	0.9690

**Table 7:** Performance improvement by using a better centroid finding method.

Sequence	Average Distance Between Our Own Centroids and the CANOPUS Centroids (Pixels)	Improved Average Great Circle Angular Distance (Arc Seconds)
8.08.2025 (38 frames)	0.27	0.7254
15.09.2025 (16 frames)	0.19	1.6358
20.10.2025, 19:54–20:01 (20 frames)	0.22	0.7659

**Table 8:** Selection of the best parameters.

Sequence	Average Image Space Speed (Pixels/Frame)	Best K	$\mathrm{Best}\;\sigma_{LoG}$	$\mathrm{Best}\;\sigma_{weight}$	Valid MEASUREMENT Points
8.08.2025 (157 frames)	0.2	7	1.0	0.8	145
20.10.2025 (66 frames)	2.5	3	6.0	0.8	59

**Table 9:** Weighted stacking vs. median stacking.

*K*	Median Stacking Valid Detection Points	$\mathrm{Weighted}\;\mathrm{Stacking}\;(\sigma_{weight}=0.8)$ Valid Detection Points
3	49	23
5	135	103
7	139	145

## Data Availability

The original images, the processed stacked images, the .tdm files and the error analysis against ground truth data for the August–September 2025 Rumba observation campaign can be downloaded from https://drive.google.com/drive/folders/1f8QbSpgZ7Htfg2RomlxAWfc0Gx6Y1-dQ?usp=sharing (Accessed on 30 January 2026).

## Abbreviations

The following abbreviations are used in this manuscript:

ESA	European Space Agency
GEO	Geosynchronous Earth Orbit
GTO	Geosynchronous Transfer Orbit
LEO	Low Earth Orbit
SNR	Signal to Noise Ratio
TLE	Two-Line Element
TDM	Tracking Data Message
RANSAC	RAndom SAmple Consensus
RA	Right Ascension Coordinate
DEC	Declination Coordinate
FWHM	Full Width at Half Maximum
